# CD28 family of receptors inter-connect in the regulation of T-cells

**Published:** 2017-09-25

**Authors:** Janna Krueger, Felix Jules, Sadiye Amcaoglu Rieder, Christopher E. Rudd

**Affiliations:** 1 Division of Immunology-Oncology, Hospital Maisonneuve-Rosemont Research Center, Montreal, Quebec H1T 2M4, Canada; 2 Department of Medicine, Université de Montréal, Montreal, Quebec H3C 3J7, Canada; 3 Medimmune, One MedImmune Way, Gaithersburg, MD 20878, USA

**Keywords:** Co-receptors, CD28, CTLA-4, PD-1, CD28H

## Abstract

T-cell activation is mediated by a combination of signals from the antigen receptor (TCR) and co-receptors such as CD28, cytotoxic T-lymphocyte antigen-4 (CTLA-4), programmed cell death antigen 1 (PD-1), CD28H and others. Each is a member of the CD28 receptor gene family. CD28 sends positive signals that promote T-cell responses, while CTLA-4 and PD-1 limit responses. It is the balance between these positive and negative signals that determines the amplitude and level of T-cell responses. The regulatory role of other family members is also becoming the focus of increasing interest. The function of certain CD28 family members such as CTLA-4 and PD-1 is dependent the expression of CD28. Together, these findings have important implications in generation of immune responses and the application of anti-receptor blocking reagents in immunotherapy.

## Introduction

### Co-receptors of the CD28 family of receptors

**Figure 1.**
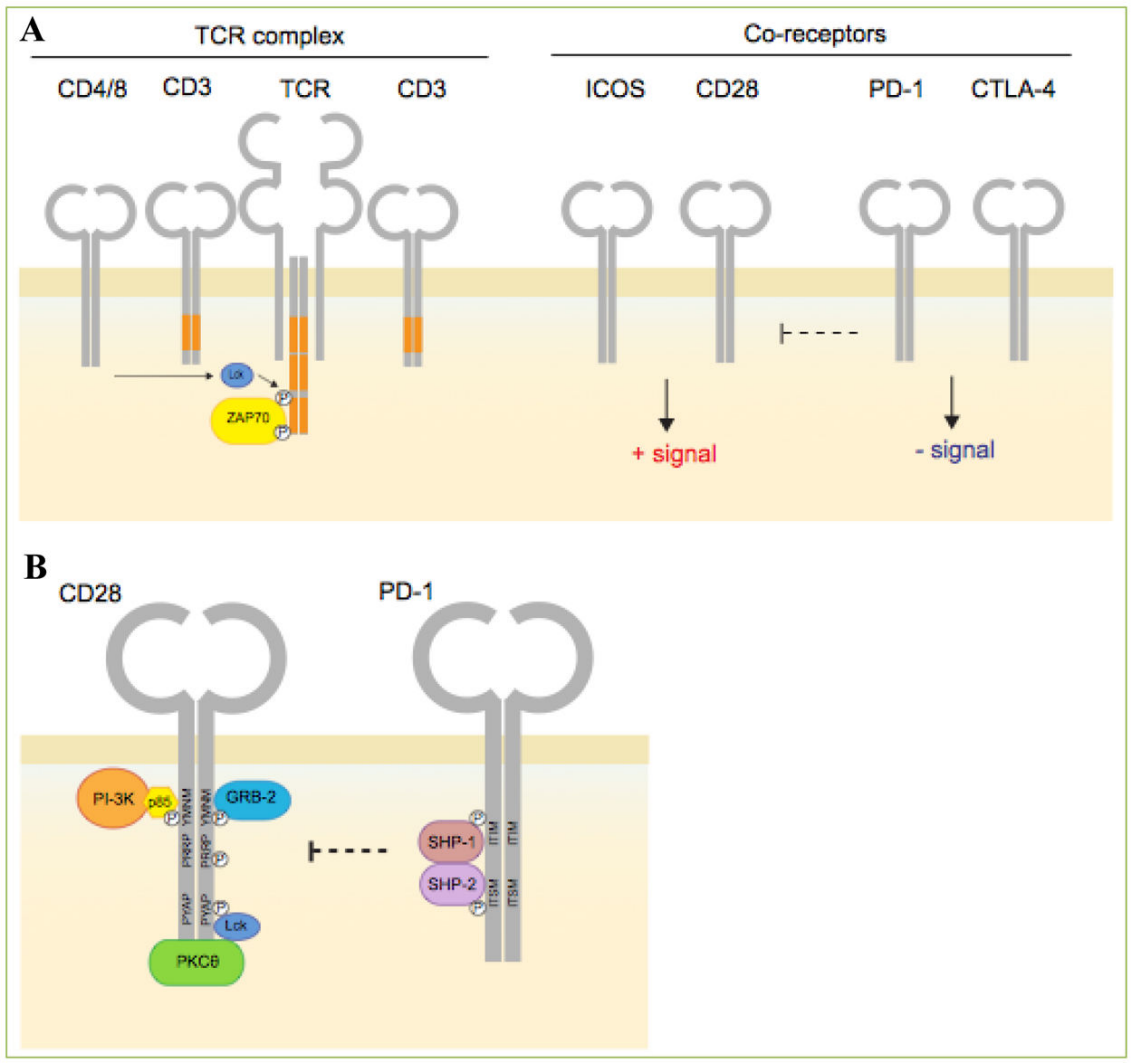
CD28 family of receptors and an interplay between PD-1 and CD28. Upper: Diagram showing the structure of the antigen-receptor (TCR) and co-receptors CD28, ICOS, PD-1 and CTLA-4 on T-cells. CD4/CD8-p56lck phosphorylates the TCR leading to the recruitment of ZAP-70. CD28 and ICOS send positive signals while PD-1 and CTLA-4 dampen the immune response. **Lower:** Illustration showing the interplay between PD-1 and CD28 at the level of signaling. p56^lck^ phosphorylates tyrosine residues on PD-1 and CD28 which allows CD28 to recruit PI 3K and GRB2, whereas PD-1 recruits the phosphatases SHP-2 (Shp2) and SHP-1 (Shp1). PI 3K binds to the CD28 pYMNM motif, while the SHP-2 (Shp2) binds to the ITIM and ITSM tyrosine motifs in PD-1. SHP2 showed a marked preference in dephosphorylating CD28 (including the pYMNM motif) relative to other substrates such as the TCRzeta chain and the co-receptor ICOS.

The co-receptor CD28 defines a family of immunoglobulin-like co-receptors and ligands involved in the regulation of the immune response. These include CD28, inducible co-stimulator (ICOS), cytotoxic T-lymphocyte antigen-4 (CTLA-4), and programmed cell death protein 1 (PD-1) (Figure 1**, upper panel**) ^[1]^. Both CD28 and CTLA-4 use their signature MYPPPY binding motifs to competitively bind ligands, CD80 (B7-1) and CD86 (B7-2) ^[2]^. CD80 binds CTLA-4 and CD28 with different affinities (K_d_ values of approximately 12 and 200 nM, respectively) ^[3]^. The higher affinity CTLA-4 binding is due to an arrangement in which bivalent homodimers bridge bivalent CD80 molecules ^[4]^. T-cells from CD28-deficient mice show reduced proliferation in response to peptide antigens ^[5, 6]^. Although high strength T-cell receptor (TCR) signaling with high avidity peptide can activate T-cells, co-ligation with CD28 is required to provide complementary signals for activation. TCR alone often results in anergy, or cell death ^[7, 8]^. Repeated antigen stimulation or long-term viral infection can bypass the requirement for CD28 ^[9]^. CD28 co-signals can stabilize cytokine messenger RNA (mRNA) ^[10]^ and play a role on cell metabolism ^[11^.

While the cytoplasmic domain of CD28 lacks intrinsic catalytic activity, it possesses key motifs that bind to SH2 and SH3 domains of signaling proteins. The src-family kinases p56lck and p59fyn phosphorylate tyrosine residues on CD28 ^[12]^. Phosphorylated YMNM binds to the SH2 domains of phosphatidyl inositol 3 kinase (PI3K) and Grb2 ^[13, 14]^. PI3K generates the lipids PIP2 and PIP3 that bind the pleckstrin homology (PH) domains within proteins such as phosphoinositide-dependent protein kinase 1 (PDK1), which in turn activates protein kinase B (PKB/AKT). The YXNX sequence of the YMNM motif binds adapter protein Grb2 ^[13, 15]^ which in turn binds exchange factor for Son of Sevenless for the activation of GTPase p21^ras^. The loss of Grb2 binding by mutation of the asparagine (N) residue leads to a loss of CD28-mediated phosphorylation of the guanine nucleotide exchange factor Vav1 and the activation of the serine/threonine kinase c-Jun kinase (JNK) ^[16]^.

### CTLA-4

Unlike CD28, CTLA-4 dampens T-cell responses in a manner that can protect against the development of auto-proliferative or autoimmune disease ^[2, 17, 18, 19]^ CTLA-4-deficient (*Ctla*-4^−/−^) mice show a profound hyper-proliferative phenotype leading to death within 3 weeks of age due to massive tissue infiltration and organ destruction ^[6]^. Antibody cross-linking is also inhibitory ^[20]^, although it is unclear whether there is a straightforward connection between these two sets of observations. Anti-CTLA-4 binds to PI3K and can activate the JNK pathway ^[21, 22]^, while concurrently inhibiting T-cell activation. CTLA-4 mediates effects via cell intrinsic and extrinsic signals ^[23, 24]^ In one model, CTLA-4 induces motility that limits contact between conventional T-cells and dendritic cells ^[25]^. Regulatory T-cells (Tregs) are resistant to this effect ^[26]^, allowing for CTLA-4 blockade of CD80/CD86 and/or removal of a portion from the cell surface ^[27]^.

### PD-1

Like CTLA-4, PD-1 is a member of the CD28 gene family that is expressed on T-cells in response to activation ^[1, 28]^. It binds to novel ligands PDL-1 (B7H-1) and PDL-2 (B7H-2) that are expressed on hematopoetic and non-hematopoetic cells. Unlike the founding member of the family, which generates positive signals that complement T-cell receptor (TCR) signaling, PD-1 produces negative signals that limit T-cell proliferation and effector functions. PD-1 inhibitory function is particularly evident in hypo-responsive ‘exhausted T-cells’ that develop with chronic viral infections and repeated antigen stimulation. Blocking antibodies against PD-1 can restore functionality to these T-cells leading to viral clearance^[29, 30, 31, 32]^.

The cytoplasmic tail of PD-1 contains two other structural motifs, an ITIM (immunoreceptor tyrosine-based inhibition, i.e. VDYGEL) motif, followed by an ITSM (immunoreceptor tyrosine-based switch, i.e. TEYSEV) motif ^[33]^ (Figure 1**, lower panel**). These motifs bind to the Src homology region 2 domain-containing phosphatase-1 (SHP-1) [also PTPN6] and related SHP-2 [PTPN11]. Both phosphatases bind to PD-1 on immune cells, although the negative signaling has been attributed to SHP-2 binding to the ITSM motif ^[1, 28, 34]^. By contrast, while CTLA-4 may associate with phosphatases, it lacks conventional binding sites, and inhibits via cell intrinsic and extrinsic mechanisms ^[24]^.

### Other family members of CD28/B7 family

Several new members of the CD28/B7 family have recently been identified. The CD28 homologue (CD28H) ^[37]^ is one such co-receptor. It is also known as the transmembrane and immunoglobulin domain-containing protein 2 variant 2 (TMIGD2) ^[35] ]^or immunoglobulin-containing and proline-rich receptor-1 (IGPR-1) ^[36]^ and shares 10% sequence homology with CD28, ICOS, CTLA-4 and PD-1 ^[37]^. CD28H is expressed in thymus, lung, heart and kidney ^[36]^ and it is constitutively expressed in all naive T-cells as well as NK cells, and is down-regulated following repeated in-vitro stimulation. Interestingly, while CD28H orthologues are present in various species, it is not present in laboratory mice or rats ^[37]^. The extracellular domain of CD28H contains an IgV-like domain and its cytoplasmic tail contains a proline rich motif that has been shown to interact with the SH3 domains of various proteins including SPIN90, CACNB2 and BPAG1. Initial studies identified CD28H as an adhesion molecule involved in cell-cell interaction, cell migration and angiogenesis ^[36, 38]^.

The ligand for CD28H has been identified as HERV-H LTR-associating 2 (HHLA2) renamed B7H7 ^[37, 39]^. Similar to CD28H, B7H7 orthologues are not present in laboratory mice or rats ^[40]^. B7H7 possesses an IgV-IgC-IgV domain in its extracellular region ^[39]^, and is expressed in intestines, breast, kidney, gallbladder, placenta and B cells ^[37, 40]^. B7H7 is believed to interact with CD28H via its first IgV domain and to send a co-stimulatory signal ^[37]^, or co-inhibitory signal in the presence or absence of CD28H expression ^[38, 41]^. Aberrant expression of B7H7 has been observed in various cancer types including lung ^[42]^ osteosarcoma ^[43]^ and pancreatic cancers ^[44]^. Other recently identified B7 family members whose overexpression correlate with poor cancer prognosis include B7H4 (B7x, B7S1, VTCN1) and B7H5 (PD-1H, VISTA, GI24, DIES1). Receptors have yet to be identified for these ligands and their functions and involvements in disease warrants further investigation.

### Immunotherapy

The past few years have witnessed breakthroughs in immune check-point blockade for the treatment of various human cancers. This was first observed with antibody blockade of the negative co-receptor CTLA-4 (cytotoxic T-lymphocyte-associated protein 4) (i.e. using Ipilimumab) and was followed by blockade of a second key co-receptor, programmed cell death-1 (PD-1) (i.e. Nivolumab and Pembrolizumab), or its ligand (PD-L1) (i.e. Atezolizumab), either alone, or in combination with anti-CTLA-4 ^[45]^.

Recent data indicates that CD28 can interface with negative co-receptors, either via extracellular or intracellular pathways. In the case of CTLA-4, studies have shown that autoimmune disease in *Ctla4*^−/−^ (i.e. CTLA-4) mice depend on CD28 expression ^[46]^. More recently, two new papers have shown that PD-1 operates via inhibition of CD28 signaling ^[47, 48, 49]^. PD-1 associates with SHP-2 which preferentially dephosphorylates CD28 (Figure 1B). The preferred de-phosphorylation of CD28 was observed in assays for reduced overall phosphorylation and PI3K SH2 domain binding to the CD28 pYMNM motif. Little de-phosphorylation of the T-cell receptor or related ICOS was observed. Both PD-1 and CD28 preferentially co-cluster to provide a mechanism by which these co-receptors interact for de-phosphorylation. Consistent with this, others have shown that PD-1 blockade requires the expression of CD28 for the recovery of exhausted T cell responses ^[48]^. The interplay involving CTLA-4 and PD-1 with CD28 may be indicative of a general theme of interplay between members of the CD28 family in the regulation of T-cell responses.

## Conclusions

The CD28/B7 family of receptors and ligands are involved in the regulation of the immune responses. Certain co-receptors such as CD28 and ICOS send positive signals while others such as CTLA-4 and PD-1 lead to a dampening of the immune response. Recent evidence indicates that this family of co-receptors interact and cross-regulate each other in regulating T-cell responses.
